# 非小细胞肺癌驱动基因检测组织标本替代品的研究进展

**DOI:** 10.3779/j.issn.1009-3419.2015.06.10

**Published:** 2015-06-20

**Authors:** 燕 孙, 正波 宋

**Affiliations:** 310022 杭州，浙江省肿瘤医院肿瘤内科 Department of Medical Oncology, Zhejiang Cancer Hospital, Hangzhou 310022, China

**Keywords:** 肺肿瘤, 基因检测, 组织标本, Lung neoplasms, Gene testing, Tumor tissue

## Abstract

基于驱动基因检测基础上的靶向药物治疗是目前晚期非小细胞肺癌的重要治疗手段。组织标本是驱动基因检测的金标准，但多数晚期肺癌患者无法获得足够的组织标本进行基因检测。探索组织标本替代品进行驱动基因检测是当前临床的热点问题。本文对驱动基因检测的组织替代标本进行综述。

## Abstract (en)

Target treatment based on driver genes in advanced non-small cell lung cancer is very important currently. Tumor tissues is the gold standard for driver genes testing. However, most of patients could not get the gene information for lack of enough tissues. To explore the tissue specimens alternatives is a hot spot in clinical work. This report reviews the tissue specimen alternatives of driver gene testing in non-small cell lung cancer.

肺癌是目前我国发病率和病死率最高的恶性肿瘤^[1]^。非小细胞肺癌（non-small cell lung cancer, NSCLC）是目前肺癌发病率最高的病理亚型，约占所有肺癌患者的80%左右，其中晚期NSCLC占新发肺癌患者的60%以上。NSCLC常见的转移部位包括肺、骨、脑及胸膜腔等。晚期NSCLC一般预后不良，中位生存时间仅约10个月-12个月左右。目前不可手术NSCLC主要的治疗手段为化疗、放疗、分子靶向治疗。其中，分子靶向治疗在延长NSCLC患者，特别是东亚、不吸烟的腺癌患者生存时间和提高生活质量方面发挥了越来越重要的作用^[2-4]^。靶向治疗的基础是驱动基因的检测，目前临床上应用较广泛的驱动基因是表皮生长因子受体（epidermal growth factor receptor, *EGFR*）、*ALK*、*ROS1*等。对于驱动基因阳性的患者，使用对应的靶向药物可使患者的中位生存时间大大延长^[5]^。

肿瘤组织标本是目前驱动基因检测的金标准，常见的获取组织标本的方式包括纵隔镜或胸腔镜活检、淋巴结活检等，但实际上多数晚期NSCLC患者往往拒绝接受创伤性的检查，因此大多数晚期NSCLC患者都无法获得足够的肿瘤标本用于检测基因突变。国内的一项多中心调查^[6]^表明，2009年中国患者*EGFR*突变检测的比例仅有9.6%，而2012年也仅仅有20%，这提示大多数患者无法获取足够的标本用于基因检测。而对于靶向药物耐药后的活检标本比例较初诊患者的标本取材率更低。IPASS研究^[2]^纳入的1, 038例患者中只有681例能够提供标本，最后仅437例患者能够评价EGFR基因突变状态。单纯的细胞学诊断仅能够满足诊断的需要，往往无法行进一步的检测。如何寻找创伤性小、有效的替代检测标本是临床中的热点问题。

## 1 外周血

目前国内外学者研究最多的是外周血检测作为组织标本的替代进行相关基因检测。肿瘤患者外周血中存在因瘤细胞凋亡、坏死而释放的游离DNA。这些游离的DNA携带了肿瘤组织中重要的驱动基因的信息。对于外周血作为驱动基因检测替代品的研究主要集中于EGFR检测方面。目前多项研究对血液中EGFR突变进行了分析。SLOG研究^[7]^是西班牙进行的一项对于存在EGFR突变者给予厄洛替尼治疗的前瞻性、Ⅱ期、多中心、单臂临床研究。在2, 105例筛选的NSCLC患者中，存在组织*EGFR*突变者有350例（16%）。其中217例接受厄洛替尼治疗，164例有配对的血清标本。结果显示，血清和组织*EGFR*突变检测的一致率为59.1%（97/164）。国内王洁等^[8]^利用变性高效液相色谱法对230例晚期NSCLC患者的配对组织和血浆*EGFR*突变的检测结果进行了分析，其中102例接受了吉非替尼治疗。两种标本的一致性为78%，该研究是迄今有关外周血与配对组织检测*EGFR*突变的最大人群的报道。2013年美国临床肿瘤学会（American Society of Clinical Oncology, ASCO）年会报道了FAST-ACT2研究通过血浆检测*EGFR*突变的结果，共224例患者的血浆进行了*EGFR*突变检测，检测方法为cobas EGFR blood test试剂盒，结果显示血浆检测和石蜡组织*EGFR*突变的阳性/阴性预测值分别为93%（68/73）和86%（130/151），总一致性为88%（198/224），血浆检测的敏感性为77%（69/90），特异性为96%（129/134）^[9]^。通过几项研究不难发现，血清或血浆用于临床*EGFR*突变检测代替组织具有一定的预测作用。当然，目前血液中检测*EGFR*突变结果与组织中的一致性方面还存在一定的问题，部分研究存在假阴性率较高，这可能会使一部分真正“阳性”的患者失去治疗的机会。目前血液作为组织标本替代品的研究多数集中在EGFR检测方面，在血液作为*ALK*、*ROS1*等基因检测方面还缺少相关的报道。血液学检测还有很多问题需要解决，如血液标本的留取时间和质控，检测的方法学等都还需要进一步探索。

循环肿瘤细胞（circulating tumor cell, CTC）是有关外周血分子标志物研究的另一焦点。其作为完整的、无损伤的肿瘤细胞，能反映肿瘤的转移、耐药、进展等过程。一项来自Dana-Farber癌症中心的研究应用微滴式数字PCR技术对血液中CTC进行肿瘤分型，探究其是否具有较高的特异性和定量能力。结果提示CTC作为肿瘤组织替代品具有良好的敏感性和特异性，且能从服用EGFR-酪氨酸激酶抑制剂（tyrosine kinase inhibitors, TKIs）药物的患者中更早地检测出T790M突变^[10]^。

2008年发表在*N Engl J Med*上的一篇文章^[11]^报道了12例晚期NSCLC的患者的外周血CTC和血浆标本，结果二者*EGFR*突变检出率分别为92%和33%，这提示我们CTC可能是更好的组织替代品。

耐药是靶向治疗不可回避的问题，也是临床诊疗的难点和热点问题。目前临床中常用的EGFR-TKIs药物及ALK抑制剂的中位有效时间约为10个月左右，绝大多数患者会出现耐药。目前耐药后的治疗还缺乏相关的标准，对于*EGFR*突变的患者耐药的原因包括T790M突变，CMET扩增、小细胞转化等^[12]^。部分耐药靶点的抑制剂已经开始进入临床研究阶段，如2014年ASCO报道的T790M抑制剂AZD9291对于靶向药物耐药后的患者可使患者的中位无进展生存期达到12个月以上。通过血浆检测T790M的情况可预测靶向药物的疗效，但血液中采用常规的PCR检测技术同样存在检测敏感性低的问题。数字PCR检测被证实敏感性高于普通PCR检测，值得进一步探索和临床应用。

## 2 胸腔积液

晚期NSCLC首诊患者约10%-15%存在恶性胸腔积液，复治患者中恶性胸腔积液的比例更高，可达到50%以上^[13]^。多项小样本回顾性研究均表明胸腔积液作为标本替代品有明显的优势：①胸腔积液多为治疗需要时获取，即仅在患者出现临床相关症状用于治疗需要时获取，因而相对容易，不增加患者额外的负担。②部分患者的胸腔积液可以反复出现，这对于动态监测患者的肿瘤的分子特征起到了重要的作用。③较血液学的检测等其他手段，胸腔积液中的肿瘤信息更加丰富。

目前已有多项临床研究显示采用胸腔积液作为组织替代品进行检测有较高的可行性。Kimura等^[14]^采用ARMS法对23例晚期肺癌患者的胸水中的肿瘤细胞进行检测显示，阳性率为35%，Zhang等^[15]^采用突变富集PCR法对胸水中的沉淀细胞和无细胞液检测，结果显示EGFR检出率为50%。Wu等^[16]^研究通过PCR方法检测136例非小细胞肺腺癌患者的恶性胸腔积液发现64%的患者存在EGFR突变，且突变率高于组织标本中*EGFR*积液突变率。Smits等^[17]^的研究表明，胸腔积液中*EGFR*突变的比例高于组织标本，但该研究采用的并非配对的组织标本。当前对于组织标本和胸腔积液中驱动基因发生率存在差异的原因可能归结于肿瘤的异质性。

目前多数关于胸腔积液中驱动基因的研究集中在*EGFR*基因检测方面，对于*ALK*融合基因的检测还缺少在胸腔积液中的数据，目前仅有来自台湾的一项回顾性研究。该研究^[18]^分析了116例EGFR阴性的患者中采用RT-PCR的方法检测*ALK*融合基因的表达情况，结果表明，共发现39例患者为ALK阳性，ALK阳性患者达到34%。通过与组织样本对比发现，其检测一致率达到85%。该研究显示RT-PCR对于胸腔积液检测具有可行性，但研究中10例患者具有配对的组织标本。而目前对于临床中开始广泛应用的其他驱动基因，如ROS1、T790M等检测的研究报道较少，是否具有可行性还缺乏研究支持。胸腔积液在靶向药物耐药的患者中出现的比例较高，可达到40%-50%左右，因此，胸腔积液作为耐药患者检测的组织替代品可能有一定的价值。但目前还缺少相关的研究。

当前对于胸腔积液中驱动基因的检测目前也存在几个问题：①目前对于胸腔积液中驱动基因的检测方法还没有确定，是采用传统的测序法还是敏感度更高的方法。②多数患者的胸腔积液为血性积液，积液中混杂的多数是血细胞，如何通过有效的方法筛选出肿瘤细胞也是当前的难点。③目前临床中多数的回顾性分析采用的是肿瘤细胞包埋形成蜡块后再行基因检测，但这无疑增加了病理工作的负担，是否可通过直接抽提积液中的游离肿瘤的DNA或RNA可以替代目前的蜡块还有待于进一步探讨。④目前的多数研究样本量较小，且均为回顾性研究，缺少胸腔积液驱动基因与组织标本配对的研究。

## 3 其他细胞学标本

由于细胞学样本中通常情况下细胞含量较少，通过敏感性较低的检测方法往往无法获取有价值的基因检测信息。随着新的敏感性更高的方法的临床应用，越来越多的研究采用细胞学样本进行基因检测。除上述提到的胸腔积液样本外，研究较多的是内镜下穿刺细胞学、脑脊液、痰液细胞学、肺泡灌洗液等样本。

Navani等^[19]^的研究纳入119例超声内镜穿刺细胞学标本，结果共107例可进行EGFR基因检测。Santis等^[20]^的研究共纳入131例超声内镜细针穿刺标本，结果126例可进行基因检测。这两项研究均提示超声内镜下细针穿刺的细胞学样本进行基因检测有较高的可行性。另外，一项临床研究^[21]^采用细针穿刺转移灶，如淋巴结、骨转移灶、气管镜刷检和冲洗液进行*EGFR*基因检测，均提示具有较高的可行性。

Yang等^[22]^的研究纳入30例NSCLC患者的脑脊液样本，采用ARMS法检测*EGFR*突变，结果共有13例患者（43%）明确为*EGFR*突变，与组织标本比较显示脑脊液作为EGFR基因检测的敏感性和特异性分别为67%和82%。

Hubers等^[23]^对晚期肺癌患者痰液中细胞DNA检测，共10例患者纳入研究，结果表明，采用不同方法检测可以发现30%-50%左右的患者存在*EGFR*基因突变，提示痰液检测可能是一个良好的替代品。

有研究者^[24]^采用肺泡灌洗液进行*EGFR*突变检测，发现有部分患者能够检测到*EGFR*突变，但研究的样本量较小，还有待于进一步探索。

## 4 展望

组织标本是晚期NSCLC基因检测的金标准，在晚期肺癌无法获取组织标本进行基因检测的情况下，尽可能的获得有价值的替代品进行驱动基因的检测具有重要的临床意义，但进一步优化检测流程，提高检测的敏感度需要临床进一步探索。在个体化治疗的时代，单纯定性检测*EGFR*及*ALK*等单一基因突变远远不够，实现定量、动态、尤其无创的基因检测才能指导更为精准的个体化治疗。目前高通量的二代测序技术已经开始在临床中使用，也有相关报道采用循环游离DNA及胸腔积液等组织替代品进行测序的相关报道，二代测序技术实现了从单基因检测到多基因检测、基因检测从定性到定量的转变，是肿瘤驱动基因检测的发展方向。
